# Case report: Experience with the Cube Navigation System in complex access routes during CT-guided lumbosacral infiltration therapy

**DOI:** 10.3389/fsurg.2023.1093964

**Published:** 2023-02-14

**Authors:** Michael Diepers, Philipp Gruber, Luca Remonda, Jatta Berberat

**Affiliations:** Department of Neuroradiology, Kantonsspital Aarau, Aarau, Switzerland

**Keywords:** computer tomography, pain, infiltration, navigation, therapy

## Abstract

**Purpose:**

Computed tomography (CT)-guided infiltrations are a mainstay in the treatment of lower back pain. Needle placement is usually performed using the free-hand method, where the translation from the planned needle angle to the actual needle insertion angle is estimated. However, the free-hand method is especially challenging in cases where a double-oblique access route (out-of-plane) rather than an in-plane route is necessary. In this case series, we report our experience with the patient-mounted Cube Navigation System to guide needle placement for complex access routes in lumbar pain therapy.

**Research design and methods:**

We retrospectively analyzed the cases of five patients in whom a double-oblique access route was necessary for CT-guided lumbar infiltration pain treatment. Each of those procedures was done using the Cube Navigation System to provide navigational guidance. The mean patient age was 69 ± 13 years (range 58–82 years; all females). Technical success, procedure time, and number of control scans were determined retrospectively.

**Results:**

Technical success (i.e., positioning and accuracy) was obtained in all cases. Mean procedure time was 15 ± 7 min (10–22 min); on average, 2 ± 1 CT control scans were performed. There were no complications or material failures reported in the present study.

**Conclusion:**

Double-oblique punctures with the Cube Navigation System in this initial case series of complex access routes at the lumbar spine were accurate and the procedure was time efficient. In the authors’ view, the Cube Navigation System has the potential to improve needle guidance for complex access routes, especially considering the ease of use of the device.

##  Introduction

Low back pain is one of the leading causes of disability and lost productivity worldwide, with an increase in disability-adjusted life years of nearly 19% from 2005 to 2015 (1). Percutaneous imaging-guided infiltrations represent one of the recommended options for minimally invasive treatment of low back pain (2–4), with computed tomography (CT) guidance used in many centers. In particular, spinal stenosis, neural foraminal stenosis, and lumbovertebral pain syndrome are commonly treated with steroid injections into the affected area (4). Accuracy of needle placement is a critical step for the success of CT image-guided percutaneous interventions as inaccurate needle placement can lead to loss of time, unwarranted radiation exposure, adverse events, or treatment failures (3).

Needle placement is most often performed with the free-hand method whereby translation from the planned needle angle to the actual needle insertion angle is estimated by the operator and confirmed *via* a series of control scans. Thus, success is dependent on the visuospatial abilities and experience of the physician. In particular, if an out-of-axial plane trajectory (double oblique) is required to achieve the best access route, needle guidance can be challenging as needle orientation becomes difficult to assess in axial control images. In these cases, CT navigation guidance systems have been shown to be helpful (3). However, many CT navigation guidance systems are impractical due to their expense, bulk, or added time required for setup (5).

A recently introduced navigation tool, the Cube Navigation System, has shown high accuracy in an *in vitro* study (6) and promising initial clinical results (7). Additionally, its relatively small size and ease of use seem to address some of the problems with other CT navigation systems. We describe our first clinical experiences using the Cube Navigation System on patients in whom a double-oblique access route was necessary.

## Case description

### Patients

We retrospectively selected from our database all cases of lumbar pain therapy where the Cube Navigation System had been used and an out-of-axial plane access was required to obtain access. Indication for lumbar infiltration therapy (either a periradicular infiltration or an epidural infiltration) was based on routine clinical workup including physical evaluation and findings from imaging studies. Ahead of treatment, all patients were screened for coagulation disorders, oral anticoagulation, platelet-inhibiting medication, and allergies. Only monotherapy with non-steroidal anti-inflammatory drug (NSAID) was allowed. All patients experienced pain-related impairment of daily activities and were assigned to us by referring clinicians after failure of non-CT-guided interventions or at least 4 weeks of conservative treatment, either with clear radicular or chronic cauda compression pain syndromes. Before infiltration planning, previous MRI or CT scans were reviewed. If findings were consistent with the diagnosis, a preliminary access route was planned.

Overall, the decision on what technique is used was at the operator's discretion. At our institution, using the Cube Navigation System may always be considered, and it is recommended for challenging access routes, particularly those not feasible in the gantry plane. Patients with a history of neurological defects are not excluded, but informed that infiltration therapy will not cure the deficit.

All patients were informed about the technique and potential complications of the lumbar injection therapy. Written informed consent was obtained from all the patients and the study was approved by the local ethical commission. See Table 1 for a timeline of care.

**Table 1:** Timeline of care.

Visit 1 (Assigning Clinician)	Visit 2 (Neuroradiology)	Visit 3 (Assigning Clinician)
Presentation of symptoms, preliminary scans, diagnosis, referral	Supplementary scans, CT-guided infiltration	Follow-up

CT, computed tomography.

### CT-guided nerve root infiltration using the Cube Navigation System

**Figure 1:**
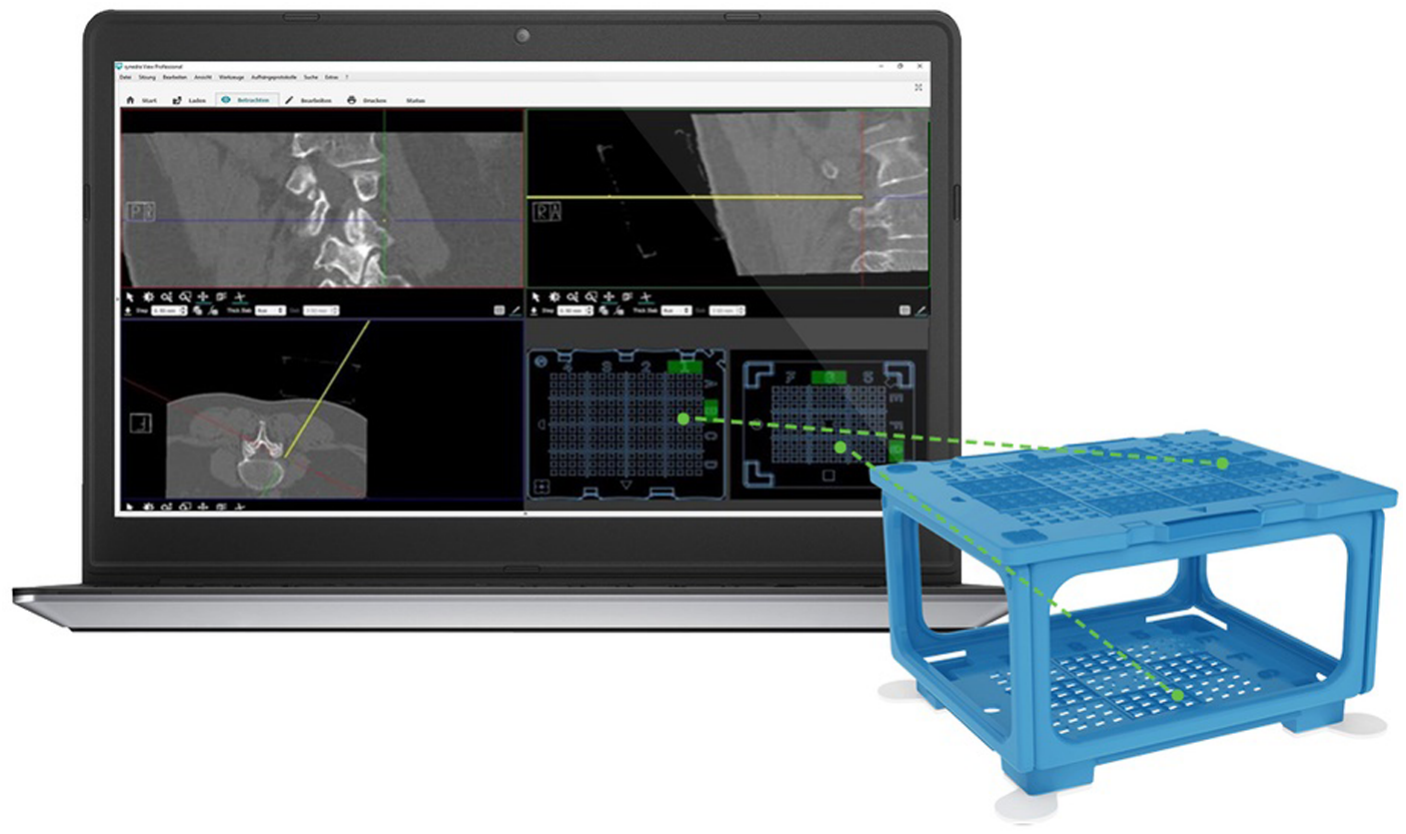
Cube Navigation System comprising of planning software shown on the computer screen on the left and scannable cube on the right. The software is displaying a lumbar infiltration with coronal, sagittal, and axial views, while the yellow line in the top right and bottom left view represents the virtual needle. The entry holes calculated from the virtual needle trajectory are indicated on the bottom right quadrant as green dots marking where the physical needle should be inserted on the top and bottom plates of the Puncture Cube in order to replicate the path of the virtual needle. (Image used with permission from Medical Templates, AG.)

The Cube Navigation System (Medical Templates AG, Egg, Zurich) consists of a patient-mounted self-adhesive cube and dedicated software that recognizes the cube in the planning scan and calculates an optimal route for the needle through the grids in the cube (Figure 1). A guidance cube is placed directly on the patient over the anticipated area of intervention using adhesive tabs and then scanned along with the patient. After firm needle insertion into the body, the cube can be collapsed (putting the upper plate on the lower plate), allowing for use of shorter needles and avoiding contact of the needle hub with the upper plate. When collapsed, slight manual needle adjustments become possible. For this study, the Puncture Cube containing openings for needles 18–22 G was used.

Patients were positioned in prone position on the CT table (Aquilion, Canon Medical Systems, Otawara, Japan). Correct placement of the Puncture Cube was guided *via* a CT topogram. After skin disinfection, the Puncture Cube was placed over the approximate puncture site and the CT planning scan was obtained during breath hold of the patient with the following parameters: 120 kV peak with 2 mm slice thickness.

**Figure 2:**
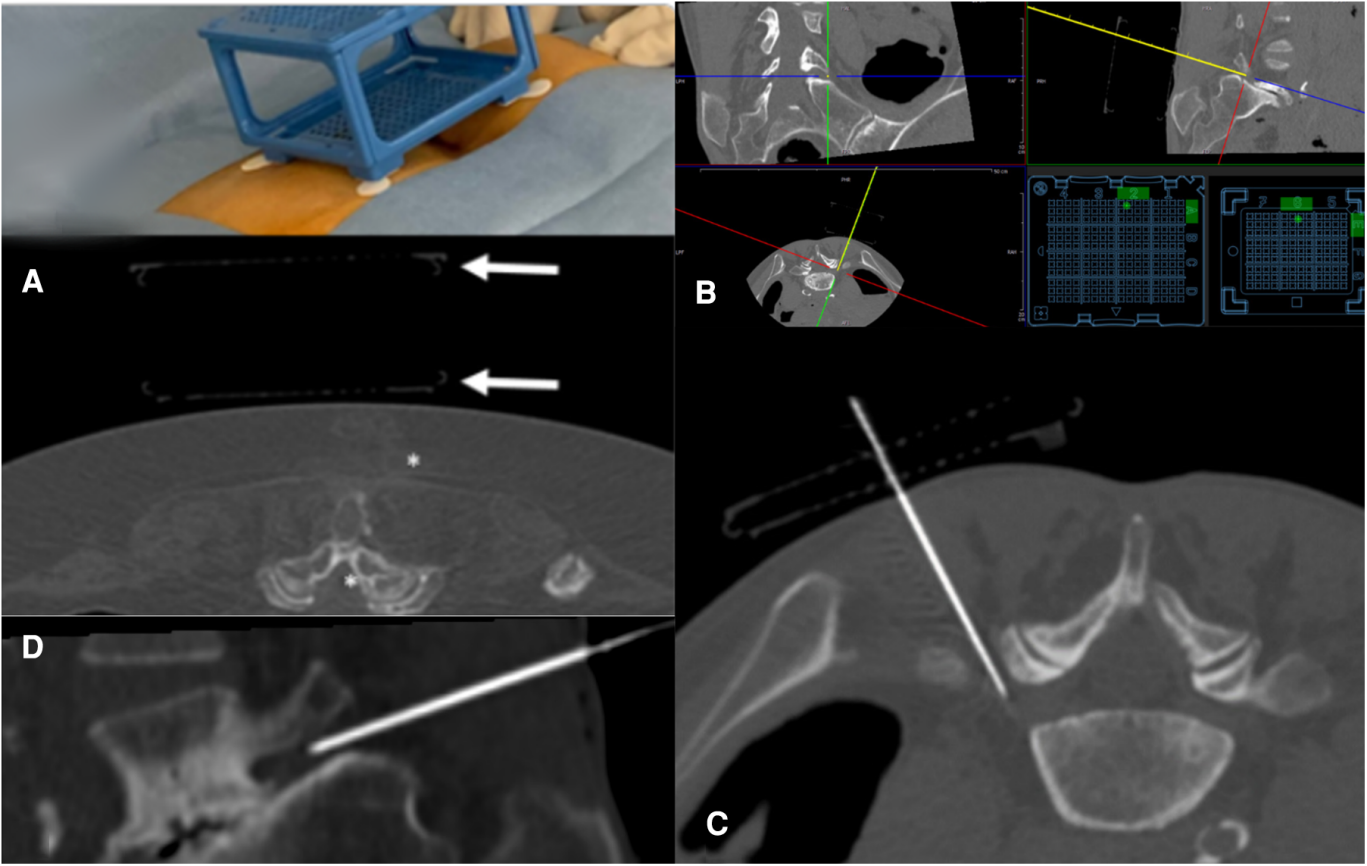
Periradicular infiltration of the L5 nerve root on the right. (**A**) On the upper right of the scan, the outline of the Puncture Cube is visible. It is fixed on the patient using the flexible pre-mounted self-adhesive tape. It is impossible to reach the L5 nerve root (asterisk) in the gantry plane. (**B**) Puncture planning with the virtual needle in craniocaudal angulation: the virtual needle is represented by the yellow dot in the top left quadrant and yellow line in the top right and bottom left quadrants. Coordinates for the Puncture Cube are represented in the bottom right quadrant by the green dots. (**C**) MPR reconstruction along the introduced needle (craniocaudal angulation) shows the now simple access path. The outline of the collapsed Puncture Cube is visible. (**D**) Sagittal MPR reconstruction along the needle path shows the angulation of the needle.

The Puncture Cube was detected relative to the patient in the planning scan using Synedra software (Synedra information technologies GmbH, Innsbruck, Austria), and a virtual model of the cube was superimposed over the scanned Puncture Cube (Figure 2). Based on a modified 3D multiplanar reformation (MPR) view, the interventionalist chose the target and could freely shift the path of a virtual needle from the target through the Puncture Cube (Figure 1C). Next, the operator introduced the puncture needle (in all cases a 20 or 22 G needle was used) into the determined coordinates in the Puncture Cube and, after a verification scan, the needle was inserted into the patient to the desired depth. In three patients, a periradicular infiltration at the level of L5 was performed, and in two patients an epidural infiltration was performed. All five procedures were performed by one experienced interventional radiologist (MD).

The procedure time (time from planning scan until the target was reached) and number of control scans (from the planning scan until the last scan with needle in place) were determined from the information in the DICOM header. In addition, it was noted whether correction of the needle path after first introduction was necessary and technical success (reaching the target without complications) was analyzed.

## Results

See Table 2 for summary of results. Mean patient age was 69 ± 13 years (range 58–82 years; all female). One patient (Pat. 2) had a history of clinically nonsuccessive decompressive surgery. In two patients (Pat. 2 and 3), fluoroscopy-guided interventions had been attempted in the referring department, albeit unsuccessfully. One patient (Pat. 5) had previous periradicular infiltrations at the level of the epidural infiltration, not performed at our institution, and not successful.

**Table 2:** Summary of results.

Patient age/sex	Diagnosis	Infiltration location	Total time (min)	Number of scans after initial planning scan
58, F	Chronic left L5 root pain syndrome due to neuroforaminal stenosis caused by spondylolisthesis and bilateral spondylolysis	L5 periradicular	13	2
82, F	Chronic lumbovertebral pain syndrome due to degenerative spinal canal stenosis	Epidural L3/4 spinal segment	22	4
67, F	Chronic left L5 root pain syndrome due to discoligament protrusion neuroforaminal stenosis	L5 periradicular	10	2
62, f	Chronic right L5 root pain syndrome due to lateral disc herniation with neuroforaminal stenosis	L5 periradicular	12	1
78, F	Chronic lumbovertebral pain syndrome due to spinal canal stenosis L2/3 caused by osteochondrosis with degenerative L2 retrolisthesis	Epidural L2/3 spinal segment	20	3

Technical success was obtained in all cases, and there were no reported adverse events. The mean procedure time to reach the target from the time point of the planning scan with the cube was 15 ± 7 (10–22) min. An average of 2 ± 1 control scans were used to reach the final target. In one patient, the first control scan required a correction of needle path after first introduction of needle as the needle path was slightly misaligned. For that, the Puncture Cube was left in place on the patient but collapsed (the upper plate was brought down to the lower plate), allowing for a slight adjustment of the needle angulation.

Two patients experienced rapid pain reduction, immediately after the infiltration (Pat. 1 and 3), while two patients experienced delayed yet sustained pain reduction (Pat. 4 and 5), and the final patient experienced a transient and incomplete effect.

## Discussion

In this small series of patients, the Cube Navigation System showed to be a promising guidance tool for percutaneous double-oblique CT-guided interventions. Needle placement proved overall to be highly accurate: in only one of the five punctures, initial needle position was slightly incorrect, most likely due to a breathing artifact. Time required for correct needle placement was well within the range when compared with the literature (7) and, in fact, the variability of procedure time appeared to be lower than that of the free-hand method from our experience. This has the potential to improve scheduling efficiency through improved estimations of procedure time.

From the patient's perspective, we assume a benefit concerning patient comfort. Usually, intervention areas with complex access routes require multiple needle corrections. In the current study, this was largely avoided. Additionally, some patients who might otherwise be considered unsuitable for the procedure due to anatomical considerations indeed may be eligible for interventional treatment using this system. In our experience, the Cube Navigation System does not add discomfort or extra time to the procedure and poses no additional risks, while potentially improving puncture accuracy and availability of treatment.

Practically speaking, the Cube Navigation System was implemented easily in the clinical routine, as only a connection from the CT modality to the Cube Navigation System software had to be installed. The lack of any space-occupying hardware equipment, as required with optical, electromagnetic, or laser systems (8–12), in the CT examination room can be regarded as an advantage to other navigation systems. Furthermore, the Puncture Cube can be used with needles of up to 18 G making it a viable option for many injections. This has promising implications for future research.

There are some limitations to the Puncture Cube. Unlike other navigation systems, larger needles cannot be used. Collapsing the cube after the initial puncture for needle correction is possible and was found necessary in one of our cases. However, it would be of advantage to have the option to remove the Puncture Cube after needle introduction completely, as otherwise the lower plate remains fixed to the patient and the scope of possible needle correction is limited. Attachment of the cube to the body by the self-adhesive tape pre-mounted on the Puncture Cube was stable at the level of the lower spine. However, at locations other than the thorax and lower back, which have essentially a plane surface, fixation might be more challenging.

Double-oblique punctures with the Cube Navigation System in this initial case series of complex access routes at the lumbar spine were accurate, and the procedure was time efficient. Prospective and comparative studies with larger patient samples for different indications and access routes are warranted for evaluation of this system.

The current case series included a limited number of patients and indications and was retrospective. While a strength of this approach is the ability to understand how the Puncture Cube performs in a limited clinical context, further studies with larger numbers of patients that are ideally prospective and comparing the Cube Navigation System to the free-hand method are necessary and planned for the future.

## Data Availability

The raw data supporting the conclusions of this article will be made available by the authors, without undue reservation.

## References

[1] Kassebaum NJ Arora M Barber RM Bhutta ZA Brown J Carter A Global, regional, and national disability-adjusted life-years (DALYs) for 315 diseases and injuries and healthy life expectancy (HALE), 1990–2015: a systematic analysis for the Global Burden of Disease Study 2015. Lancet. (2016) 388(10053):1603–58. 10.1016/S0140-6736(16)31460-X 27733283 PMC5388857

[2] Filippiadis DK Kelekis A. A review of percutaneous techniques for low back pain and neuralgia: current trends in epidural infiltrations, intervertebral disk and facet joint therapies. Br J Radiol. (2016) 89(1057):20150357. 10.1259/bjr.20150357 26463233 PMC4985947

[3] Begemann PGC. CT-guided interventions: indications, technique, and pitfalls. In: Mahnken A Wilhelm KE Ricke J, editors. CT- and MR-guided interventions in radiology. Berlin: Springer (2013). p. 11–24.

[4] Manchikanti L Knezevic NN Navani A Christo PJ Limerick G Calodney AK Epidural interventions in the management of chronic spinal pain: American Society of Interventional Pain Physicians (ASIPP) comprehensive evidence-based guidelines. Pain Physician. (2021) 24(S1):27. 10.36076/ppj.2021.24.s27-s208 33492918

[5] Chehab MA Brinjikji W Copelan A Venkatesan AM. Navigational tools for interventional radiology and interventional oncology applications. Semin Intervent Radiol. (2015) 32(4):416–27. 10.1055/S-0035-1564705 26622105 PMC4640922

[6] Mokry A Willmitzer F Hostettler R Richter H Kircher P Kneissl S Evaluation of a novel, patient-mounted system for CT-guided needle navigation-an ex vivo study. Neuroradiology. (2018) 61(1):55–61. 10.1007/s00234-018-2107-0 30506482

[7] Grigoriadis S Filippiadis D Stamatopoulou V Alexopoulou E Kelekis N Kelekis A. Navigation guidance for percutaneous splanchnic nerve radiofrequency neurolysis: preliminary results. Medicina. (2022) 58(10):1359. 10.3390/MEDICINA58101359 36295520 PMC9607001

[8] Schubert T Jacob AL Pansini M Liu D Gutzeit A Kos S. CT-guided interventions using a free-hand, optical tracking system: initial clinical experience. Cardiovasc Intervent Radiol. (2013) 36(4):1055–62. 10.1007/s00270-012-0527-5 23232857

[9] Putzer D Arco D Schamberger B Schanda F Mahlknecht J Widmann G Comparison of two electromagnetic navigation systems for CT-guided punctures: a phantom study. RoFo. (2016) 188(5):470–8. 10.1055/s-0042-103691 27074422

[10] Durand P Moreau-Gaudry A Silvent AS Frandon J Chipon E Médici M Computer assisted electromagnetic navigation improves accuracy in computed tomography guided interventions: a prospective randomized clinical trial. PLoS One. (2017) 12(3):e0173751. 10.1371/journal.pone.0173751 28296957 PMC5351986

[11] Gruber-Rouh T Schulz B Eichler K Naguib NNN Vogl TJ Zangos S. Radiation dose and quickness of needle CT-interventions using a laser navigation system (LNS) compared with conventional method. Eur J Radiol. (2015) 84(10):1976–80. 10.1016/j.ejrad.2015.07.004 26210096

[12] Kettenbach J Kronreif G. Robotic systems for percutaneous needle-guided interventions. Minim Invasive Ther Allied Technol. (2015) 24(1):45–53. 10.3109/13645706.2014.977299 25421786

